# Still SDAPing Along: 20 Years of the Structural Database of Allergenic Proteins

**DOI:** 10.3389/falgy.2022.863172

**Published:** 2022-03-22

**Authors:** Catherine H. Schein, Surendra S. Negi, Werner Braun

**Affiliations:** ^1^Department of Biochemistry and Molecular Biology, Institute for Human Infections and Immunity, University of Texas Medical Branch at Galveston, Galveston, TX, United States; ^2^Sealy Center for Structural Biology and Molecular Biophysics, University of Texas Medical Branch at Galveston, Galveston, TX, United States; ^3^Margaret Maccallum Gage and Tracy Davis Gage Professorship in Biochemistry and Allergies, University of Texas Medical Branch at Galveston, Galveston, TX, United States

**Keywords:** allergenic protein nomenclature, sequence and structure, physicochemical property scale, IgE epitopes, history of allergen studies, peanut and nut allergens, property distance scale, component resolved extracts

## Abstract

The introduction of plant extracts to mitigate the symptoms of “hay fever”, about a century ago, led to discoveries beginning sixty years ago on determining the sequences and eventually structures of allergenic proteins. As more proteins were cloned, there was a need to rapidly identify and categorize those with significant similarity to known allergens. The Structural Database of Allergenic Proteins (SDAP) was created at the beginning of the 21st century as the first cross-referenced website to allow rapid overview of the structures and sequences of allergenic proteins. SDAP provides a way to identify sequence and functional similarities between these proteins, despite the complex nomenclature system based on the Latin names of their different sources. A rapid FASTA search simplifies grouping allergens from the same structural or functional family. SDAP also provides an overview of the rapidly expanding literature on the sequence, structure and epitopes of allergenic proteins and a way to estimate the potential allergenicity of novel proteins based on rules provided by the IUIS. Twenty years and a pandemic later, the list of allergenic proteins and their attributes continues to grow. SDAP is expanding and improving to allow rapid access to all this information.

## Introduction: Naming Allergens

This report marks 20 years since the online version of the Structural Database of Allergenic Proteins (SDAP) was first established. SDAP's original purpose was to provide a cross referenced website to classify allergens according to their names, structure and function, a need that had been building for over 100 years. Although allergic reactions are described in print as early as the 16th century, or even ancient times (1, 2), the word allergy, and the beginning of treatments for allergy with plant extracts, began in the first years of the 20th century (3). Extracts of ragweed helped many individuals with “hay fever,” but injecting patients with whole pollen extracts could also induce dangerous anaphylaxis. Thus researchers, aided by modern protein chemistry, set out to make safer, simpler, “component resolved” extracts. Molecular studies of the protein components in the extracts that could contribute to reactivity began about 60 years ago. During the standardization of these extracts, it was found that there were several components that bound IgE in sera from hypersensitized individuals, leading to modern tests for categorizing the types of allergens a patient might react to (4). The first isolated allergenic proteins, Amb a 1 and Amb a 2 of ragweed (5, 6) were soon followed by many others from many different sources.

A standardized nomenclature based on the Latin names of their plant, animal, insect or venom source was first published in 1984 (7). The nomenclature they agreed upon was to designate highly purified allergenic proteins by the first three letters of the genus followed by a space, the first letter of the species name, followed by a space, both italicized, followed by a Roman numeral to indicate the order of importance (or isolation) of the protein. For example, the first perennial rye grass allergen, from the plant with the Latin name *Lolium perenne* was called *Lol p* I.

The list of proteins in the paper, mostly aeroallergens from dander and pollen, covered less than a journal page. The only “ingested” allergens were parvalbumin from cod (Gad c 1), three egg white proteins (Gal d 1-3) and a surface protein of round worm (Asc s 1).

**Figure 1:**
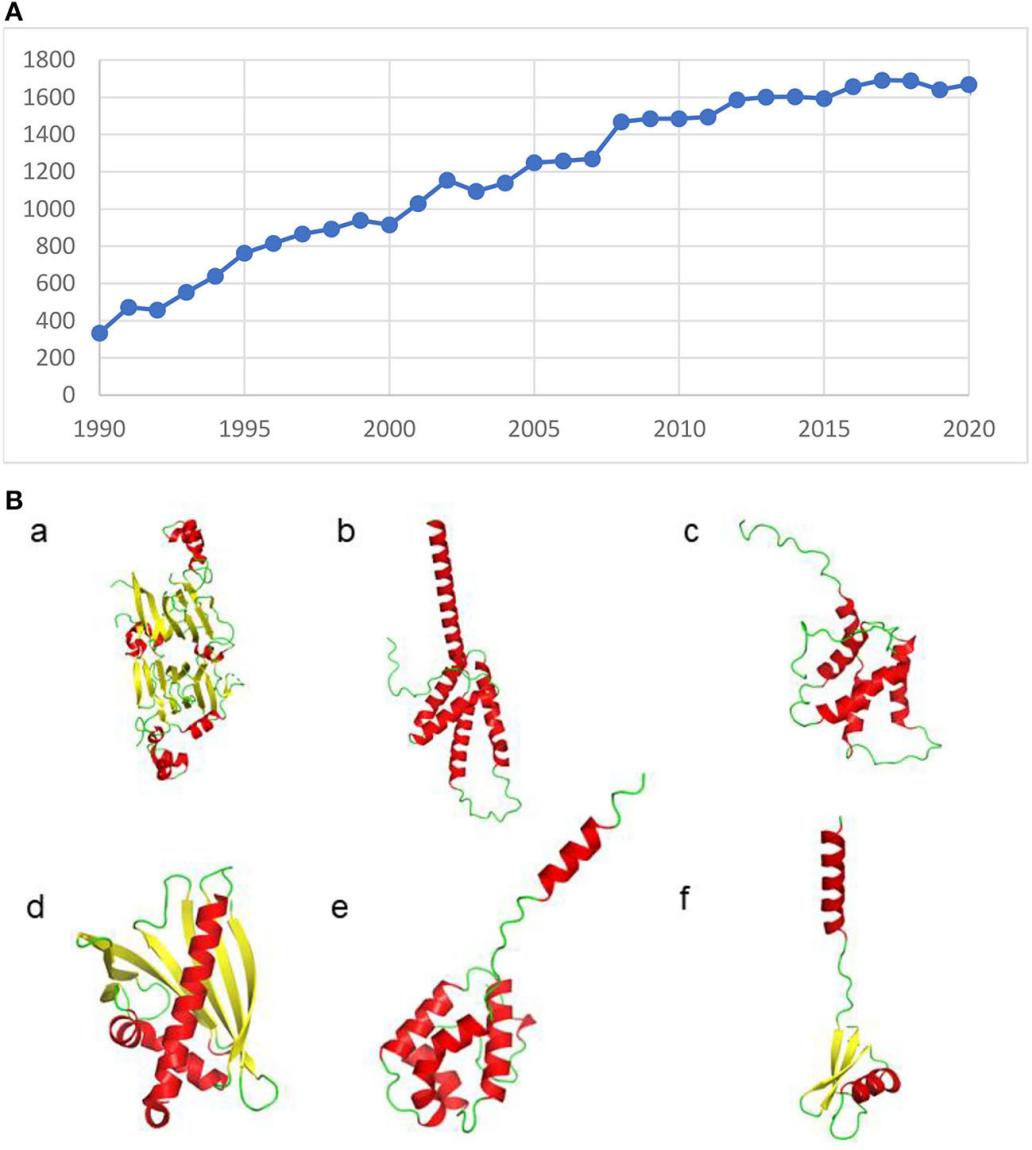
**(A)** Publications per year with the words Allergenic Proteins in Pubmed are steadily increasing. **(B)** Allergens have diverse structures, as ribbon structures of some of the allergens identified in peanuts show. a) Ara h 1 (PDB id: 2SMH), b) Ara h 2 (PDB id: 2OB4), c) Ara h 6 (PDB id: 1W2Q), d) Ara h 8 (PDB id: 4M9B) e) Ara h 9 and f) Ara h 12. The loop region in Ara h 2 and structures of Ara h 9 and Ara h 12 were modeled using Alphafold.

The allergen field expanded rapidly (some called it a “data explosion”), thanks to innovations in immunology, protein sequencing and the recognition that IgE in serum bound specifically to allergens (Figure 1). Multiple allergens were isolated from peanuts (8) and other sources (9, 10).

The nomenclature was simplified a few years later (11), in that names were no longer italicized, and Arabic, not Roman numbers were used (e.g., Ara h 8 from peanuts instead of VIII). The list of allergens in the 1994 paper stretched over more than four journal pages, with additional recommendations, not just for the sequences of the whole protein, but for peptides from the sequence that were epitopes for IgE from patient sera. But numbering remained a problem. The small table in the 1984 publication shows that Api m I, Ves g I, Pol a I all clearly referred to phospholipases of honey bee, yellow jacket and wasps, respectively, while Api m II, Ves g II, and Pol a II refer to hyaluronidases. But new allergens of different families, such as pectate lyases and from various pathogen related (PR) groupings (12, 13) were named as they were isolated, so the numbers no longer reliably predicted similarity in function or structure. For example, the vicilin allergen of peanut, Ara h 1, corresponded best in structure to Jug r 2 of walnut. Later identified allergens, Ara h 6 and Ara h 7, had similarity to Ara h 2 and other 2S albumins.

### Defining Allergens for Inclusion Into SDAP

The first question in assembling SDAP was which proteins should be included. For clinicians, the word allergen refers simply to a food or pollen the patient reacts to, such as milk, shrimp, peanut or ryegrass. SDAP's goal is to aid researchers or regulators who need a more molecular definition by cataloging all the proteins or protein fragments that contribute to the allergenicity of the plant or animal source (which is specified for all entries). For inclusion, SDAP relies primarily on the WHO/IUIS list provided at their website (http://allergen.org/), as these proteins have been reviewed by a committee of experts in the field. Due to potential anaphylactic reactions in direct assays, such as patch testing or oral food challenge, most proteins are classified as allergenic if they bind IgE from sera of a sufficient number of patients with clinically diagnosed reactivity to the source. However, to quote from the WHO/IUIS website:

“The primary goal of a systematic nomenclature is to define a common language for scientists. As such, *assessment of new allergen candidates for inclusion into this database does not involve a judgement on their clinical significance* (my italics). A minimal criterion of demonstrated IgE binding to the suggested allergen using sera from patients allergic to the specific source is required.”

In addition to the IUIS list, other proteins have been included if they were listed in one of the existing data bases containing allergens [see Table 1 in (14) and for a more recent discussion of databases, see (15)]. These “non-IUIS” entries (clearly marked as such) are kept in SDAP as a service for researchers who are exploring and studying proteins that might have a potential allergenic response. Some proteins of wheat that cause non-IgE mediated symptoms in sensitive individuals have also been included, again to help those seeking to define the potential relationship between these proteins and known allergenic ones. The files in SDAP contain more information and literature references for highly studied component proteins, such as those of peanut or shrimp. Additional literature searching may be needed for the less studied and especially “non-IUIS” proteins.

## SDAP Guide to Allergenic Proteins

The first job of SDAP was thus to provide a cross-referenced list of the sequences and associated information of all the proteins acknowledged to be allergenic by the IUIS (16). This was done by a series of cross referenced MySQL lists, most of which were assembled tediously by human effort (17). Later versions of the database could use automatic identification (14), but many proteins were found to be allergenic or IgE_binding only after their isolation and naming.

The need for bioinformatic tools to identify such potential allergens was brought to the forefront by an attempt to enhance the methionine content of grains, by inserting a newly identified gene from a brazil nut (18, 19). These projects were terminated when allergic responses were found to the “genetically modified” foods. Thus, a sequence FASTA search in SDAP was implemented to rapidly show whether a test protein had significant identity to any known allergen, using a set of rules established by the IUIS (20). The user could then decide whether to proceed with using the protein or drop projects before problems arose.

### Identifying Areas Similar to Known IgE Epitopes

Many allergens known to cross react, such as those from peanut and walnut, have very low sequence identity. The next SDAP innovation, the peptide similarity scale, found similar sequences using a physicochemical property (PCP) scale of the amino acids determined by the Braun group (21, 22). The scale was first used to identify common motifs (23), similar regions within allergens, that could be used to identify potentially cross reacting epitopes (24) even in allergens with very different structures (25–29). Other webserver or downloadable tools can be used to further analyze SDAP results. Episearch maps peptide mimotopes from phage libraries to allergenic proteins (30) and DGraph allows one to view the “property distances (PD)” between, for example, IgE reactive sequences or whole related protein sequences, as a 2D-map, without an initial sequence alignment (27, 31, 32).

### Structures for All Allergens

Allergens can have many diverse structures (Figure 1B) (33–37) with functional (38) or even “disordered” (39) regions that contain epitopes. One of the most important and distinguishing features of SDAP is the incorporation of structural data, through direct links to files in the Protein Database (PDB) or model structures made from suitable templates (34, 36).

## Conclusions

The information on allergens' structures and epitopes continues to grow at a rapid rate. SDAP was created to help understand the similarities and differences in these proteins. Twenty years after its start, there is now a major push to update its software and list of allergenic proteins and their isomers, to be a tool for researchers, regulatory agencies and patients.

## Author Contributions

CHS wrote and edited the manuscript with help from WB. SN prepared Figure 1B. All authors contributed to the article and approved the submitted version.

## Funding

SDAP was originally funded by a Research Development Grant (#2535-01) from the John Sealy Memorial Endowment Fund for Biomedical Research, grants from the US-Food and Drug Administration (FD-U-002249), and the Texas Higher Education Coordinating Board (ATP grant 004952-0036-2003) to WB. Our work with allergenic proteins and SDAP upkeep has been supported by grants from the U.S. Environmental Protection Agency under STAR Research Assistance Agreements (RD-833137, RD-834823-01 to WB, RE-83406601 to CHS), the US Department of Agriculture (ARS 58-6435-9-40 to CHS), the National Institute of Health (R01 AI 064913 to WB, 1R01AI165866-01 to Stephen Dreskin; subcontract to CHS), and funding for this article was provided by the Margaret Maccallum Gage and Tracy Davis Gage Professorship in Biochemistry and Allergies to WB.

## Conflict of Interest

The authors declare that the research was conducted in the absence of any commercial or financial relationships that could be construed as a potential conflict of interest.

## Publisher's Note

All claims expressed in this article are solely those of the authors and do not necessarily represent those of their affiliated organizations, or those of the publisher, the editors and the reviewers. Any product that may be evaluated in this article, or claim that may be made by its manufacturer, is not guaranteed or endorsed by the publisher.
